# Association of *ACE2* Gene Variants with Adverse Perinatal Outcomes in COVID-19 Infected Pregnant Women in Kazakhstan

**DOI:** 10.3390/v16111696

**Published:** 2024-10-30

**Authors:** Kymbat Mukhtarova, Karina Tazhibayeva, Aigul Myrzabekova, Vitaliy Koikov, Zaituna Khamidullina, Milan Terzic, Gauri Bapayeva, Saule Zhumambayeva, Azliyati Azizan, Antonio Sarría-Santamera

**Affiliations:** 1 Department of Biomedical Sciences, School of Medicine, Nazarbayev University, Astana 010000, Kazakhstan; kymbat.mukhtarova@nu.edu.kz; 2 Department of Obstetrics and Gynecology #1, NJSC “Astana Medical University”, Astana 010000, Kazakhstan; 3 Department of Surgery, School of Medicine, Nazarbayev University, Astana 010000, Kazakhstan; milan.terzic@nu.edu.kz; 4 Clinical Academic Department of Women’s Health, CF “University Medical Center”, Astana 010000, Kazakhstan; 5 College of Osteopathic Medicine, Touro University, Henderson, NV 89014, USA

**Keywords:** COVID-19, SARS-CoV-2, ACE2, SNP, low birthweight, preterm birth

## Abstract

SARS-CoV-2 utilizes the angiotensin-converting enzyme 2 (ACE2) receptors located on membranes to enter host cells. Nevertheless, the *ACE2* gene primarily encodes for a zinc metalloproteinase, which is a part of the renin–angiotensin system (RAS). ACE2 downregulation results in the deregulation of RAS in favor of pro-fibrosis, pro-apoptosis, oxidative stress, pro-inflammation, aldosterone production and release, and blood vessel contraction axis. ACE2 is highly expressed in the placenta. There are both axes of the RAS system in the placenta. This study aims to assess the perinatal outcomes with ACE2 receptor polymorphisms in pregnant women infected with SARS-CoV-2 during pregnancy. The case-control study was conducted to determine the association of *ACE2* single-nucleotide polymorphisms in 171 COVID-19-positive pregnant subjects and 112 control subjects. The recessive mutations of rs2158082 and rs4830974 were associated with an increased risk of low birthweight and preterm birth, whereas the dominant mutation of rs2285666 (CT + TT) was associated with decreased odds of low birthweight. COVID-19 was not a significant factor contributing to the adverse perinatal outcomes in our sampling. These findings may help to clarify the controversy regarding the increased risk of adverse perinatal outcomes observed during COVID-19 as well as provide new perspectives for research on the genetic factors associated with a higher risk of adverse perinatal outcomes.

## 1. Introduction

The outbreak of the novel coronavirus disease caused by the SARS-CoV-2 virus (COVID-19) that began in the Hubei Province, Wuhan, in China was declared a pandemic in March 2020 by the World Health Organization (WHO).

The virus utilizes the angiotensin-converting enzyme 2 (ACE2) receptors to enter the host cells. ACE2 is highly expressed in the colon, kidney, central nervous system, respiratory tract, cardiac fibroblasts, cardiomyocytes, and vascular smooth muscle cells. Spike protein is the major protein of SARS-CoV-2 used to invade the cells expressing ACE2 receptors [1].

Classically, the *ACE2* gene is known to code for a zinc metalloproteinase, which is primarily a part of the renin–angiotensin system (RAS). The RAS is responsible for blood pressure control via the ACE–AngII–AT1 axis. This axis favors pro-fibrosis, pro-apoptosis, oxidative stress, pro-inflammatory responses, aldosterone production and release, and blood vessel contraction. However, there is a balancing protective mechanism via the alternative ACE2-Ang-(1-7)–MAS axis [2]. Consequently, one can hypothesize that the ACE–AngII–AT1 arm is activated by ACE, and if the ACE2 protein is unavailable, there is no counterbalance to its consequences. ACE2 downregulation by SARS-CoV-2 results in the deregulation of RAS in favor of the ACE–AngII–AT1 axis. According to Gómez and colleagues [3], the RAS signaling pathway is very strongly associated with hypertension, diabetes, and cardiovascular disease, and the balance of ACE1 and ACE2 activity is essential for the pathogenesis of diseases including respiratory diseases. Interestingly, ACE2 also plays a critical role in pregnancy; its expression is upregulated to compensate for preeclampsia via the modulation of angiotensin 1-7.

However, there has been a significant concern based on both individual research and systematic reviews regarding the impact of viral epidemics on the course of pregnancy [4]. The role of SARS-CoV-2 infection on perinatal outcomes is not clear and yet there are conflicting views on the impact of COVID-19 on pregnancy outcomes [5,6]. ACE2 receptors are highly expressed in the placenta, especially in the syncytiotrophoblasts, cytotrophoblasts, endothelial cells, and vascular smooth muscle cells [7]. Syncytiotrophoblasts are part of the innate immune system as they are a physical barrier to infections [8]. The study of Patanè et al. [9] found viral Spike proteins in the fetal side of the placenta. Despite this, intrauterine vertical transmission of SARS-CoV-2 is not common. In turn, both infection of syncytiotrophoblasts of the placenta and feto–maternal vascular malperfusions are prevalent [7]. It has been reported that COVID-19 increases the odds of preterm birth [10,11,12,13,14,15] and low birthweight [11,12]. Likewise, increased rates of fetal distress, cesarean delivery, and both iatrogenic and spontaneous preterm births have been associated with COVID-19 [4]. Inhibition of the ACE2 receptors could potentially lead to the pathologic vasculature of the placenta, affecting the gas and nutrient feto–maternal exchange. Thus, this study aims to assess the possible associations of perinatal outcomes with ACE2 receptor polymorphisms in women infected with SARS-CoV-2 during pregnancy.

## 2. Materials and Methods

### 2.1. Study Design

A case-control study was conducted to determine the association of single-nucleotide polymorphisms (SNPs) in the *ACE2* gene. The inclusion criteria for cases included being diagnosed with COVID-19 during pregnancy, with a SARS-CoV-2 infection confirmed through real-time polymerase chain reaction (RT-PCR). The control group comprised of pregnant women without COVID-19 during pregnancy and with no diagnosed infectious diseases or other health concerns. There was no exclusion based on the ethnicity of the participants. Cases were recruited from November 2021 to February 2022. Control subjects were recruited from September to October 2023. In total, data from 283 females were analyzed for the study. All participants were recruited from the Multifunctional City Hospital #3, Astana, Kazakhstan. Participants’ blood samples and demographic and clinical records were collected by medical personnel who have access to the medical records. Then, retrospective data about pregnancy outcomes were obtained from the above-mentioned medical records. The outcome measures were low birthweight and preterm birth. Namely, low birthweight is defined as the weight at birth of less than 2500 g. Preterm birth is the birth of alive infants earlier than 37 weeks of gestation. All study participants provided written informed consent. Ethical approval was obtained from the Nazarbayev University Institutional Research Ethics Committee (NU-IREC) #745/12062023.

### 2.2. Genetic Data

Whole blood samples of participants were de-identified and collected in EDTA-containing vacutainer tubes by the medical personnel of Multifunctional City Hospital #3 perinatal center. DNA was extracted by using the Wizard Genomic DNA Purification Kit (Promega, Madison, WI, USA) according to the manufacturer’s protocol. Quantitation and quality of DNA were ascertained using a NanoDrop 2000 spectrophotometer (Thermo Fisher Scientific, Wilmington, DE, USA). Genotyping was performed using qualitative real-time PCR (Bio-Rad, Hercules, CA, USA) in 384-well plates. Thermal cycling conditions were as follows: polymerase activation at 95 °C for 10 min, followed by 40 cycles of denaturation (at 95 °C for 15 s) and annealing extension (at 60 °C for 1 min).

### 2.3. Statistical Analysis

Data cleaning was performed using Microsoft Excel. All statistical analyses were conducted using the Stata 14.2 (Stata Corporation, College Station, TX, USA) statistical program and SNPStats online tool based on R [16]. Basic descriptive statistics, such as frequencies and mean values, were generated. To assess the association with the outcome variable, Fisher’s exact test was used for categorical independent variables, and the Wilcoxon rank-sum test was used for continuous independent variables. To estimate the strength of the association between the polymorphisms and COVID-19, multivariate logistic regression analysis was performed. Demographic covariates were included in the adjusted model to adjust for their possible confounding effect on the outcome variable. The odds ratio (OR) and 95% confidence interval (CI) were calculated. Linkage disequilibrium and haplotype analysis were conducted. Participants were divided into the following two groups: cases (patients with preterm birth/patients with low birthweight) and subsequent controls. All statistical tests were two-sided. Following the Bonferroni correction or multiple comparisons, *p* < 0.017 was taken as significant for the *ACE2* polymorphisms within the genetic associations analysis. A significance level (α) equal to 0.05 was chosen for descriptive statistics. The Hardy–Weinberg equilibrium test and bivariate statistics for the different inheritance patterns were conducted as well.

## 3. Results

### 3.1. Demographic Data

A total of 283 pregnant women were recruited for this study. Up to 60.4% of them had lab-confirmed COVID-19 during pregnancy. Table 1 represents the sociodemographic and clinical characteristics of the women. The groups were matched by age (*p* > 0.05). Clinical characteristics such as total days hospitalized, levels of leukocytes, lymphocyte level, international normalized ratio (INR), prothrombin time, protein, creatinine, and ALT were statistically significantly different. Most patients had a moderate severity of COVID-19. The worst respiratory rate was 18.78 ± 1.62. The mean oxygen saturation was 98.23 ± 0.95. Almost 60% of the patients required oxygen supplementation and almost 40% of the sampling of COVID-19 patients had ARDS.

### 3.2. Perinatal Outcomes

Table 2 demonstrates variations in the characteristics of the cases with adverse perinatal outcomes and controls. There is no prominent effect of SARS-CoV-2 infection on low birthweight (*p* = 1.000) and preterm delivery (*p* = 0.815). However, it is possible to notice the following in cases with low birthweight and preterm births: higher creatinine, INR, and ALT levels; lower erythrocyte, prothrombin, protein, and urea levels; lower Apgar scores; lower birthweight and gestational age; higher frequency of C-sections; and more cases of induced labor. Age, antenatal care registration, gravidity, and parity were not statistically significantly different between the cases and the controls (Table 2). Table S1 shows the bivariate associations of the pregnant women’s demographic, clinical characteristics, and perinatal outcomes with low birthweight and preterm birth. It reports the statistically significant negative association of preterm birth with the levels of monocytes, erythrocytes, and Apgar scores. There are increased odds of preterm birth with higher levels of INR, C-sections, and induced labor. C-sections and induced labor are also associated with approximately five times the odds of a low birthweight. Similarly, a sufficient level of erythrocytes and lower levels of urea are linked to lower odds of low birthweight in our sampling (Table S1). There are associations of the three adverse perinatal outcomes with gestational age and birthweight, but they are more likely to be consequences. As for the COVID-19-associated factors in only the COVID-19 sampling (171 pregnant women), low birthweight was present in 15 and preterm birth was present in 14 patients. However, none of the COVID-19-associated factors were statistically significantly associated with adverse outcomes (Table S1).

### 3.3. Genetic Association Study

Overall, three ACE2 SNPs (rs2158082, rs4830974 and rs2285666) were genotyped. All of them were in Hardy–Weinberg equilibrium (Table S2) and thus eligible for further analysis.

**Figure 1:**
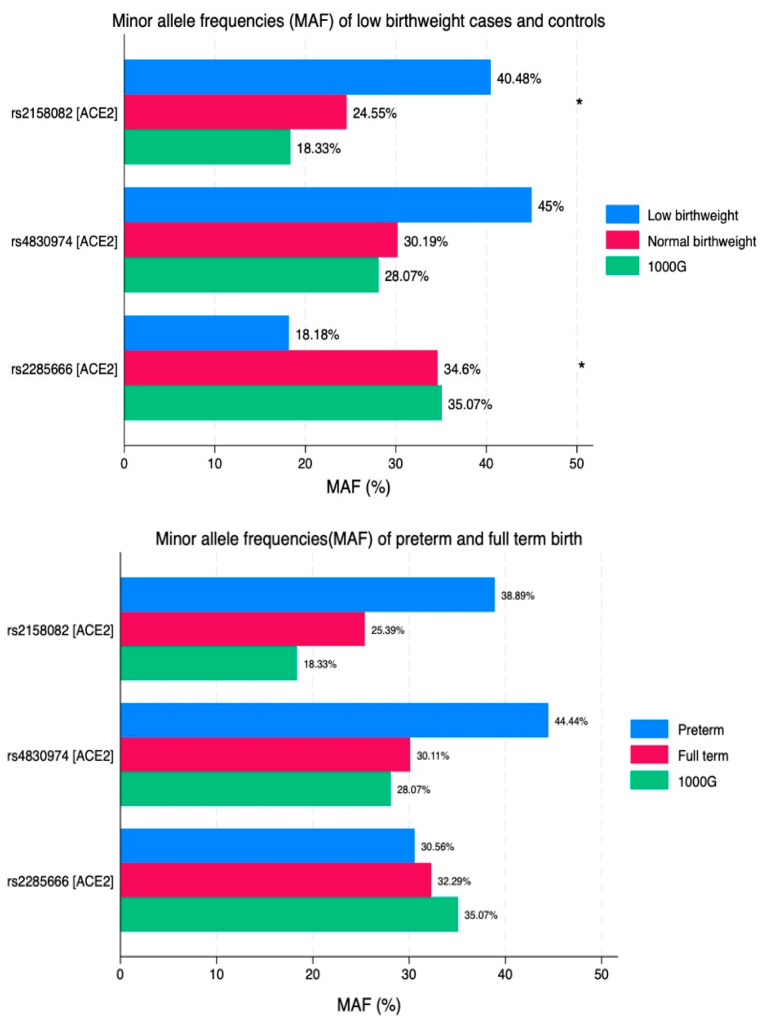
Minor allele frequencies of ACE2 SNPs in adverse maternal and perinatal outcomes. * Indicates *p* < 0.05.

Figure 1 demonstrates the distribution of minor allele frequencies (MAFs) in the cases and in the controls and compares them with the MAFs in the general population (1000G). There is a statistically significant difference in rs2158082 (A > G) in women giving birth to low birthweight infants, which is *p* = 0.04. The rs2285666 (C > T) mutation is almost two times lower in the low birthweight cases than in the controls (*p* = 0.029); it is also lower than in the general population (1000G).

The unadjusted odds ratios of maternal and perinatal outcomes (Table S2) in five modes of inheritance show that the recessive mode of inheritance of rs2158082 and rs4830974 are better suited for investigating relationships with low birthweight. rs2285666 in the dominant mode resulted in the lowest AIC/BIC, which was 145.5/152.51. Applying the same principle, to examine the associations of the SNPs with preterm birth, it is more suitable to use the recessive modes of rs2158082 and rs4830974, and the dominant mode of rs2285666.

Linkage disequilibrium showed a certain level of deviation from the expected genotype frequencies in our sampling (D’ ≠ 0), and it is valid for combinations of all three SNPs (LD *p*-value < 0.05) (Figure S1). This highlights the importance of considering co-segregation at these sites.

Table 3 demonstrates the haplotype analysis in three SNPs of *ACE2* gene. From haplotype analysis, CGG (block 3) was found to be statistically significantly associated with the odds of a low birthweight (*p* = 0.000) regardless of COVID-19 status. Haplotype CGG also showed a strong association with preterm birth. Namely, there is an approximately 3-fold (3.05 (1.45–6.44)) increase in the odds of a preterm birth in women conferring the G allele in rs2158082 (A > G), G allele in rs4830974 (A > G), and C allele of rs2285666 (C > T), and this is statistically significant (*p* = 0.003). Also, COVID-19 was shown to increase the odds of preterm birth by roughly 20% in CGG haplotype.

**Figure 2:**
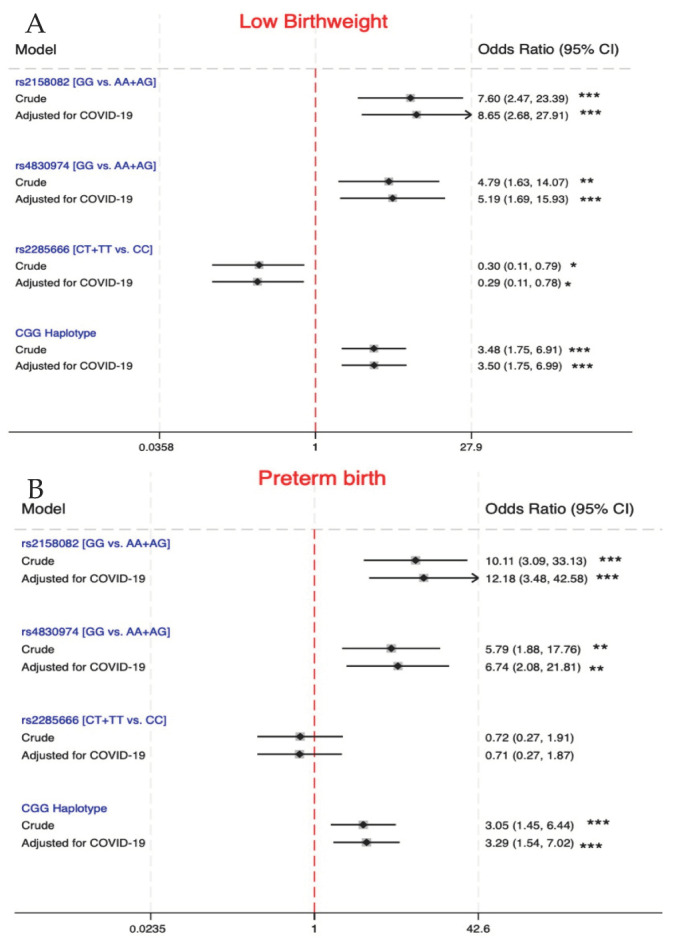
Association of candidate SNPs with maternal and perinatal outcomes: (**A**) with low birthweight and (**B**) preterm birth. * indicates *p* < 0.017; ** indicates *p* < 0.0085; *** indicates *p* < 0.00425.

Figure 2 reports the crude and adjusted odds ratios of the three outcomes as well as the ACE2 gene polymorphisms and CGG haplotype. GG vs. AA + AG mutations in rs2158082 and rs4830974 are associated with an increase in the odds of a low birthweight (Figure 2A). In the presence of a SARS-CoV-2 infection, crude OR increases from 7.6 (2.47–23.39) to 8.65 (2.68–27.91) for rs2158082; and from 4.79 (1.63–14.07) to 5.19 (1.69–15.93) for rs4830974. rs2285666 resulted in no change in the odds of a low birthweight. To be exact, regardless of COVID-19 status, the dominant mode of inheritance of rs2285666 resulted in approximately 0.3 (0.11–0.79) times the odds of low birthweight in pregnant women with the CT + TT genotype in comparison with the wild-type CC genotypes. CGG in pregnant women is associated with an increase in the odds of low birthweight (Figure 2A). Crude OR increases from 3.48 (1.75–6.91) to 3.5 (1.75–6.99) in COVID-19-infected pregnant women, which is not significantly different from the crude OR.

In terms of preterm birth, the homozygous mutant GG of rs2158082 and homozygous mutant GG of rs4830974 are associated with higher odds of a preterm birth (Figure 2B). In contrast, rs2285666 was not statistically significantly associated with the risk of a preterm birth (*p* > 0.017). COVID-19 during pregnancy increased the odds of a preterm birth in rs2158082 and rs4830974 but not in rs2285666. The odds of a preterm birth in CGG haplotype carriers are 3.05 (1.45–6.44) times higher than in females with other variants in the three SNP sites. Interestingly, COVID-19 increases the odds of a preterm birth by approximately 24% in CGG haplotype carriers.

## 4. Discussion

This study has examined the associations of ACE2 SNPs and COVID-19 with adverse perinatal outcomes in two groups of pregnant women from a single hospital in Kazakhstan. To date and the best of our knowledge, this is the first study assessing this association. The main finding of this study is that COVID-19 did not represent an independent effect on adverse perinatal outcomes. Moreover, none of the COVID-19-associated factors were statistically significantly associated with adverse perinatal outcomes in our sampling. However, individual genetic variations of the ACE2 gene were found to be associated with low birthweight and preterm birth. Since all three genotypes (rs2285666, rs2158082, and rs4830974) are under strong linkage disequilibrium, the CGG haplotype was found to be associated with higher odds of low birthweight and preterm birth.

Preterm birth has been identified as a frequent complication affecting 9–39% of pregnancies that tested positive for COVID-19 [6]. In our sample, it was present in 9.33% of the COVID-19 cases and 7.53% of the healthy controls. Preterm birth and low birthweight were more likely in pregnant women with severe SARS-CoV-2 sickness than in those with moderate disease [11].

On the other hand, a reduction in low birthweight infants in the general population was observed during the COVID-19 lockdown [17,18]. However, these changes have been explained by favorable socio-environmental factors related to the introduction of pandemic-related non-pharmaceutical restrictions rather than the contribution of clinical or molecular factors, including fewer working hours, reduced stress of work, increased family support, a reduced load of infections, and governmental financial support [13,18]. Improvements in preterm births may, however, be occurring in high-income countries [19], while low- and middle-income countries may have experienced significant reductions in maternal services [20].

It is complex to compare our findings with comparable studies as there is no evidence of genetic studies conducted to investigate the associations of ACE2 polymorphisms with adverse perinatal outcomes in COVID-19 patients.

Nevertheless, we can compare our findings with the other studies conducted to examine the effect of SNPs on ACE2 expression. Namely, the rs2285666 variants may influence ACE2 expression via alternative splicing mechanisms [21]; people with the AA (TT) genotype had ACE2 expression levels over 50% lower than in people with the GG (CC) genotype [22]. Wild-type CC genotype of rs2285666 was reported to be associated with higher susceptibility to COVID-19, mortality, and severity [21,23]. Both rs4830974 and rs2158082 are eQTL variants of ACE2 and are known to upregulate the expression of ACE2 [22].

Our study has shown that the dominant C allele (increased expression of ACE2) of rs2285666 is associated with higher odds of low birthweight. This can be explained by the fact that higher levels of ACE2 expression on cell surfaces can lead to high chances of SARS-CoV-2 infections. We demonstrated that SNPs associated with the higher expression of ACE2 are associated with adverse perinatal outcomes.

Tamanna et al. [24] reported that higher ACE2 activity drives higher levels of Ang-(1-7), and Ang-(1-7) is lower in women with preeclampsia. Moreover, early gestation elevations of ACE and ACE2 are observed in small gestational age pregnancies [24]. A possible explanation of the conflicting views about the effect of ACE2 expression on pregnancies is that there can be a protective mechanism by a soluble form of ACE2 (sACE2). Namely, elevated sACE2 can inhibit the binding of SARS-CoV-2 to a membrane-bound form of ACE2 and thus protect against the invasion of SARS-CoV-2 into the tissues [24]. Moreover, ACE2 knockout mice were shown to have higher blood pressure during pregnancy and gave birth to growth-restricted offspring [25].

Interestingly, immune cells (circulating granulocytes, monocytes, and lymphocytes) also express ACE2 and thus can serve as a reservoir for SARS-CoV-2 [26]. Nevertheless, there is very limited evidence of vertical transmission of COVID-19 [27], but a healthy pregnancy depends on immune cells located at the decidua. They help to remodel the uteroplacental circulation during the first trimester [26]. According to Hamilton and colleagues [28], labor is associated with the increased macrophage infiltration of the decidua. Macrophages, in turn, activate the production of proinflammatory cytokines and prostaglandins [28]. Moreover, the invasion of the placenta by ACE2-expressing neutrophils and macrophages increases the risk of fetal distress [29].

COVID-19 can impact the exchange of nutrients in the placenta, and the placenta was characterized as having both arms of RAS system [25]. The placentas from SARS-infected women in the third trimester showed extensive fetal thrombotic vasculopathy and areas of avascular fibrotic villi [8,30], with more maternal vascular malperfusions (MVMs) [31,32], fetal vascular malperfusions (FVMs), and intervillous thrombi [31]. MVMs are associated with preterm birth, fetal growth restriction, and fetal demise, whereas FVMs are associated with fetal vascular thrombosis, abnormal cord insertion, and the hyper coiling of the umbilical cord. Chen and colleagues reported that massive perivillous fibrin deposition (MPVFD) is characteristic of a SARS-CoV-2 infection [33]. MPVFD is known to be associated with intrauterine fetal death, prematurity, and fetal growth restriction [34], and it is a rare condition [35].

An important aspect is the spatial–temporal distribution of ACE2 in the placenta during pregnancy. In early pregnancy, ACE2 is more concentrated in the decidua, in the area surrounding the villi, whereas a low content of ACE2 is found at the feto–maternal interface. As gestation advances, there is a change in the ACE2 protein expression patterns, and higher expressions are found in the syncytiotrophoblast, villous endothelial cells, and cytotrophoblasts. These changes in ACE2 distribution suggest the placental susceptibility to SARS-CoV-2 and that the disease manifestation may vary throughout gestation [6]. Despite the evidence of the significant role that ACE2 plays in pregnancy, the exact mechanisms regulating and mediating ACE2 expression and function in pregnancy are still unknown. Lower levels of ACE2 protein in placentas from COVID-19-positive pregnancies have also been reported [36], suggesting that SARS-CoV-2 infection may directly or indirectly change ACE2 expression and biological functions in pregnant women. Furthermore, it is known that placental hypoxia is associated with overexpression of ACE2 [27].

Overall, because ACE2 acts as an entry door for SARS-CoV-2 infection, there may be an interruption of ACE2’s physiological function. This could be a main factor contributing to the increased risks of complications and disease in mothers and children exposed to COVID-19 during pregnancy, including preterm birth, low birthweight, preeclampsia, or small for gestational age.

Our study has some limitations. It is a study analyzing a restricted sample conducted in a single healthcare center. The participants in our study were a convenience sample of pregnant women with COVID-19, as either spontaneous demand or referred to the center, which could leave more vulnerable groups underrepresented. Because the participants were recruited in 2021, in the early phases of the pandemic, there were no homogeneous protocols/ICU admission criteria for these patients. Patients hospitalized for COVID-19 were treated with different drugs (corticosteroids, lopinavir/ritonavir, azithromycin, hydroxychloroquine, interferon beta1, tocilizumab, and prophylactic or therapeutic heparin). In the period analyzed here, vaccination was not authorized for pregnant women in Kazakhstan. The period covered included the second wave of COVID-19 cases in the country mostly related to the Delta variant. Delta was known to be more pathogenic to the placenta in comparison with the alpha and omicron variants [37]. The impact on pregnant women vaccinated or infected by other variants could not be derived from the above-mentioned data.

## 5. Conclusions

The study analyzed the clinical and demographic variables and ACE2 genetic profiles of pregnant women infected with SARS-CoV-2, along with the uninfected controls. These results suggest that women infected with COVID-19 had comparable perinatal outcomes to those not infected during pregnancy; however, individual ACE2 genetic characteristics have a significant effect on pregnancy outcomes, as the recessive mutations of rs2158082 and rs4830974 were found to be associated with an increased risk of low birthweight and preterm birth, whereas the dominant mutation of rs2285666 (CT + TT) was associated with decreased odds of low birthweight. In turn, COVID-19 minimally contributes to the associations but does not act as a significant effect modifier. These findings may help to clarify the controversy over whether a COVID-19 infection represents an increased risk of perinatal outcomes in pregnant women, as well as provide new perspectives for research on the genetic factors associated with a higher risk of adverse perinatal outcomes. Future studies should explore the role of circulating plasma ACE2 levels. Circulating ACE2 vs. membrane-bound ACE2 expression can help to explain the existence of conflicting views about COVID-19’s role on pregnancy outcomes.

## Figures and Tables

**Table 1:** Demographic and clinical characteristics of pregnant women with COVID-19 during pregnancy and controls.

Variables	COVID-19 (*n* = 171)	Control (*n* = 112)	Total (*n* = 283)	Exact *p*-Value
Sociodemographic characteristics of pregnant women				
Age	29.49 ± 6.27	29.47 ± 5.71	29.48 ± 6.05	0.809
Kazakh ethnicity	156 (91.76%)	97 (86.61%)	253 (89.72%)	0.168
Days spent in hospital	6.5 ± 2.28	4.73 ± 2.5	5.81 ± 2.52	0.000 ***
Primigravida	50 (29.24%)	18 (16.07%)	68 (24.03%)	0.015 *
Multipara	82 (47.95%)	88 (78.57%)	170 (60.07%)	0.000 ***
Antenatal care (ANC) registration	129 (90.85%)	85 (93.41%)	214 (91.85%)	0.626
Biochemical and immunological characteristics of pregnant women				
Hemoglobin	112.17 ± 14.38	113.95 ± 12.96	112.85 ± 13.86	0.609
Leukocytes	7.92 ± 2.67	10.21 ± 2.86	8.79 ± 2.96	0.000 ***
Neutrophils	72.52 ± 11.97	74.77 ± 1.76	72.97 ± 9.71	0.530
Lymphocytes	21.8 ± 8.84	18.75 ± 6.05	20.98 ± 7.95	0.041 *
Monocytes	6.32 ± 3.19	5.48 ± 1.39	5.98 ± 2.64	0.111
Platelets	243.72 ± 64.31	241.84 ± 65.74	243.00 ± 64.74	0.606
Erythrocytes	3.98 ± 0.49	4.04 ± 0.43	4.00 ± 0.47	0.433
APTT	28.81 ± 4.49	28.63 ± 3.65	28.75 ± 4.21	0.777
INR	0.97 ± 0.77	0.94 ± 0.1	0.96 ± 0.62	0.000 ***
Fibrinogen	3.87 ± 1.05	3.95 ± 0.58	3.89 ± 0.93	0.076
Prothrombin	11.2 ± 3.27	11.66 ± 0.81	11.37 ± 2.66	0.005 **
Protein	65.34 ± 9.00	69.72 ± 6.02	66.23 ± 8.65	0.001 **
Bilirubin	8.02 ± 4.17	8.69 ± 4.72	8.22 ± 4.34	0.157
Urea	2.59 ± 0.89	2.96 ± 0.82	2.66 ± 0.89	0.003 **
Creatinine	66.18 ± 14.94	55.6 ± 10.51	64.09 ± 14.77	0.000 ***
ALT	20.8 ± 29.08	18.43 ± 28.04	20.3 ± 28.82	0.011 *
AST	23.79 ± 20.17	22.09 ± 17.09	23.43 ± 19.54	0.082
Perinatal and current pregnancy related characteristics				
Preeclampsia	3 (1.75%)	4 (4.08%)	7 (2.60%)	0.261
Hypertension	12 (7.02%)	14 (14.29%)	26 (9.67%)	0.057
Apgar 1 min	7.74 ± 0.9	7.69 ± 1.13	7.72 ± 1.00	0.974
Apgar 5 min	8.6 ± 1.00	8.63 ± 1.37	8.61 ± 1.17	0.034 *
C-section	41 (27.15%)	17 (16.04%)	58 (22.57%)	0.048 *
Urinary Tract Infection	38 (22.49%)	4 (4.04%)	42 (15.67%)	0.000 ***
Induced labor	23 (19.49%)	16 (19.51%)	39 (19.50%)	1.000
Gestational age at delivery (weeks)	38.63 ± 2.6	39.18 ± 2.01	38.89 ± 2.34	0.049 *
Birthweight of fetuses (grams)	3299.25 ± 730.51	3410.18 ± 632.07	3344.75 ± 692.7	0.223
Current COVID-19 associated factors				
Severity at admission				
Mild	15 (9.62%)	-	-	-
Moderate	139 (89.10%)	-	-	-
Severe	2 (1.28%)	-	-	-
The worst respiratory rate	18.78 ± 1.62	-	-	-
Oxygen saturation the worst	98.23 ± 0.95	-	-	-
Oxygen supplementation	102 (59.65%)	-	-	-
Acute respiratory distress syndrome (ARDS)	67 (39.18%)	-	-	-
Symptoms				
Fever	106 (61.99%)	-	-	-
Anosmia	13 (7.60%)	-	-	-
Sweating	18 (10.53%)	-	-	-
Headache	86 (52.76%)	-	-	-
Sore throat	107 (65.64%)	-	-	-
Runny nose	95 (58.28%)	-	-	-
Nasal congestion	116 (71.17%)	-	-	-
Cough	133 (81.60%)	-	-	-
Muscle weakness	156 (81.23%)	-	-	-
Myalgia	59 (34.50%)	-	-	-
Diarrhea	2 (1.17%)	-	-	-
Vomiting	14 (9.27%)	-	-	-
Shortness of breath	38 (22.22%)	-	-	-
Chest discomfort	38 (22.22%)	-	-	-
Pneumonia	55 (32.16%)	-	-	-
Treatment during COVID-19 hospitalization and prescriptions during current pregnancy				
Remdesivir	22 (12.87%)	-	-	-
Antibiotics	142 (83.04%)	-	-	-
Bronchodilators	70 (40.94%)	-	-	-
Anticoagulants	148 (86.55%)	-	-	-
Antiplatelet	12 (7.14%)	-	-	-
Mucolytics	2 (1.17%)			
Corticosteroid therapy (Dexamethasone)	94 (54.97%)	-	-	-
Hepatoprotective therapy	77 (45.03%)	-	-	-
Paracetamol	52 (30.77%)	-	-	-
Antianemic	43 (26.22%)	-	-	-
Antiemetic	5 (3.05%)	-	-	-

* Indicates *p* < 0.05; ** indicates *p* < 0.01; *** indicates *p* < 0.001; “-“denotes “not applicable”. Note: continuous variables are presented as (mean ± SD), and categorical variables are given as (*n*, %).

**Table 2:** Demographic, clinical, and perinatal characteristics of low birthweight and preterm birth in cases vs. controls.

Variables	Low Birthweight			Preterm Delivery		
	Case (*n* = 24)	Control (*n* = 259)	Exact *p*-Value	Case (*n* = 21)	Control (*n* = 262)	Exact *p*-Value
Sociodemographic characteristics of pregnant women						
Age	29.75 ± 5.27	29.46 ± 6.12	0.842	30.14 ± 5.62	29.5 ± 6.1	0.699
Kazakh ethnicity	20 (83.33%)	233 (90.31%)	0.288	16 (76.19%)	201 (90.54%)	0.058
Days spent in hospital	6.67 ± 3.37	5.73 ± 2.41	0.347	6.14 ± 2.71	5.63 ± 2.42	0.480
Primigravida	6 (25.00%)	62 (23.94%)	1.000	5 (23.81%)	53 (23.87%)	1.000
Multipara	16 (66.67%)	154 (59.46%)	0.523	14 (66.67%)	132 (59.46%)	0.643
Antenatal care (ANC) registration	18 (90.00%)	196 (92.02%)	0.671	16 (100.00%)	171 (90.00%)	0.372
Biochemical and immunological characteristics of pregnant women						
Hemoglobin	110.79 ± 17.81	113.04 ± 13.45	0.698	109.4 ± 19.27	113.4 ± 12.74	0.290
Leukocytes	9.39 ± 3.7	8.73 ± 2.88	0.558	9.06 ± 4.38	8.83 ± 2.89	0.534
Neutrophils	73.1 ± 8.79	72.95 ± 9.83	0.993	72.56 ± 10.42	73.35 ± 10.05	0.812
Lymphocytes	20.89 ± 7.54	20.99 ± 8.00	0.963	19.83 ± 8.51	20.76 ± 8.08	0.543
Monocytes	5.91 ± 2.98	5.98 ± 2.61	0.626	5.88 ± 2.92	6.01 ± 2.64	0.887
Platelets	246.83 ± 57.96	242.64 ± 65.45	0.708	229.35 ± 57.53	245.24 ± 65.9	0.290
Erythrocytes	3.8 ± 0.6	5.44 ± 22.44	0.04 *	3.64 ± 0.54	4.02 ± 0.46	0.001 ***
Activated partial thromboplastin time (APTT)	29.43 ± 3.61	28.68 ± 4.27	0.323	28.81 ± 4.49	28.63 ± 3.65	0.777
International normalized ratio (INR)	0.92 ± 0.08	0.96 ± 0.65	0.270	0.97 ± 0.77	0.94 ± 0.1	0.000 ***
Fibrinogen	3.92 ± 1.55	3.89 ± 0.85	0.560	3.87 ± 1.05	3.95 ± 0.58	0.08
Prothrombin	10.97 ± 2.51	11.41 ± 2.67	0.905	11.2 ± 3.27	11.66 ± 0.81	0.005 **
Protein	64.78 ± 16.14	66.37 ± 7.6	0.556	65.34 ± 9.00	69.72 ± 6.02	0.001 **
Bilirubin	7.56 ± 3.45	8.3 ± 4.43	0.654	8.02 ± 4.17	8.69 ± 4.72	0.157
Urea	2.72 ± 0.74	2.66 ± 0.9	0.431	2.59 ± 0.89	2.96 ± 0.82	0.003 **
Creatinine	60.73 ± 11.97	64.44 ± 15.02	0.286	66.18 ± 14.94	55.6 ± 10.51	0.000 ***
Alanine transaminase (ALT)	18.55 ± 11.02	20.48 ± 30.07	0.508	20.8 ± 29.08	18.43 ± 28.04	0.011 *
Aspartate aminotransferase (AST)	21.15 ± 6.95	23.67 ± 20.39	0.779	23.79 ± 20.17	22.09 ± 17.09	0.082
COVID-19	15 (62.5%)	156 (60.23%)	1.000	14 (66.67%)	136 (61.26%)	0.815
Perinatal and current pregnancy related characteristics						
Preeclampsia	2 (8.33%)	5 (2.04%)	0.122	0 (0.00%)	6 (2.78%)	1.000
Hypertension	2 (8.33%)	24 (9.8%)	1.000	0 (0.00%)	24 (11.11%)	0.236
Hyperglycemia	0 (0.00%)	8 (3.27%)	1.000	0 (0.00%)	7 (3.24%)	1.000
Apgar 1 min	6.27 ± 1.88	7.86 ± 0.74	0.000 ***	7.74 ± 0.9	7.69 ± 1.13	0.974
Apgar 5 min	7 ± 1.93	8.77 ± 0.94	0.000 ***	8.6 ± 1.00	8.63 ± 1.37	0.034 *
Gestational age at delivery	34.96 ± 5.01	39.29 ± 1.38	0.000 ***	38.63 ± 2.6	39.18 ± 2.01	0.049 *
Birthweight	1806.19 ± 611.6	3503.91 ± 470.17	0.000 ***	3299.25 ± 730.51	3410.18 ± 632.07	0.223
C-Section	13 (54.17%)	45 (19.31%)	0.000 ***	9 (42.86%)	46 (20.72%)	0.029 *
Gestational Diabetes	0 (0.00%)	5 (2.04%)	1.000	0 (0.00%)	5 (2.31%)	1.000
Urinary Tract Infection	4 (16.67%)	38 (15.57%)	0.776	2 (10.00%)	35 (16.20%)	0.748
Induced Labor	8 (57.14%)	31 (16.76%)	0.002 **	6 (46.15%)	31 (17.51%)	0.022 *

* Indicates *p* < 0.05; ** indicates *p* < 0.01; *** indicates *p* < 0.001. Note: continuous variables are presented as (mean ± SD), and categorical variables are given as (*n*, %).

**Table 3:** ACE2 haplotypes’ associations with perinatal outcomes.

Haplotype Analysis							
Outcome	Block	rs2285666	rs2158082	rs4830974	Case/Control	OR (95% CI)	AOR (95% CI)
Low birthweight	1	C	A	A	0.4000/0.4598	Ref.	Ref.
	2	T	A	A	0.1579/0.2521	0.56 (0.23–1.37)	0.56 (0.22–1.37)
	3	C	G	G	0.4000/0.1608	3.48 (1.75–6.91) ***	3.5 (1.75–6.99) ***
Preterm birth	1	C	A	A	0.3235/0.4767	Ref.	Ref.
	2	T	A	A	0.2258/0.2378	0.93 (0.39–2.26)	0.94 (0.39–2.26)
	3	C	G	G	0.3824/0.1686	3.05 (1.45–6.44) ***	3.29 (1.54–7.02) ***

*** indicates *p* < 0.00425; indicates *p* < 0.05. AOR—adjusted for COVID-19 status. Note: haplotypes with total frequencies less than 5% were eliminated from analysis.

## Data Availability

All data generated or analyzed during this study are included in this article and its additional files. The row genotyping data used and/or analyzed during this study are available from the corresponding author upon reasonable request.

## Supplementary Materials

The following supporting information can be downloaded at: https://www.mdpi.com/article/10.3390/v16111696/s1, Table S1: Bivariate analysis of patient characteristics with maternal or perinatal complications; Table S2: Association of ACE2 polymorphisms with maternal and perinatal outcomes. Genotype frequencies and inheritance patterns; Figure S1: Linkage disequilibrium of the ACE2 SNPs.
