# Supplementary material for: Association of ACE2 Gene Variants with Adverse Perinatal Outcomes in COVID-19 Infected Pregnant Women in Kazakhstan
Source: Viruses. 2024 Oct 30;16(11):1696. doi: 10.3390/v16111696 (PMC11598833; doi:10.3390/v16111696)
Supplement: Supplementary file 1 [file viruses-16-01696-s001.zip › viruses-3255344-supplementary.pdf]

Supplemental materials

Table S1. Bivariate analysis of patient characteristics with maternal or perinatal complications.

Variables	Low birthweight (n=24)	Preterm birth (n=21)	PROM (n= 16)
Age	1.01 (0.94 – 1.08)	1.02 (0.95 – 1.1)	0.99 (0.91 – 1.07)
Kazakh ethnicity	0.54 (0.17 – 1.69)	0.33 (0.11 – 1.00)	1.68 (0.21 – 13.3)
Days spent in hospital	1.15 (0.98 – 1.34)	1.09 (0.91 – 1.29)	0.8 (0.63 – 1.01) ¶
Primigravida	1.06 (0.4 – 2.79)	1.00 (0.35 – 2.85)	1.06 (0.33 – 3.43)
Multipara	1.36 (0.56 – 3.3)	1.36 (0.53 – 3.51)	2.2 (0.69 – 7.01) ¶
Hb	1.00 (0.97 – 1.03)	1.00 (0.97 – 1.03)	0.99 (0.95 – 1.02)
Leukocytes	1.10 (0.97 – 1.26) ¶	0.93 (0.78 – 1.10)	**1.24 (1.07 – 1.43) ****
Neutrophils	1.02 (0.95 – 1.09)	0.96 (0.89 – 1.04)	1.02 (0.96 – 1.08)
Lymphocytes	1.00 (0.94 – 1.05)	0.99 (0.93 – 1.05)	0.97 (0.9 – 1.04)
Monocytes	0.86 (0.70 – 1.06) ¶	**0.72 (0.55 – 0.94)***	0.84 (0.67 – 1.06) ¶
Platelets	0.99 (0.99 – 1.00) ¶	0.99 (0.99 –1.00) ¶	1.00 (1.00 – 1.01)
Erythrocytes	**0.39 (0.17 – 0.91) ***	**0.21 (0.08 – 0.56) ****	0.74 (0.26 – 2.15)
Activated partial thromboplastin time (APTT)	1.02 (0.92 – 1.13)	0.97 (0.86 – 1.09)	1.02 (0.9 – 1.17)
International normalised ratio (INR)	0.40 (0.01 – 13.35)	**26.01 (2.24 – 301.39)****	0.11 (0.00 – 14.73)
Fibrinogen	0.69 (0.37 – 1.29) ¶	1.33 (0.8 – 2.2)	0.85 (0.45 – 1.63)
Protrombin	0.91 (0.78 – 1.06) ¶	1.01 (0.84 – 1.22)	0.96 (0.8 – 1.16)
Protein	1.03 (0.97 – 1.10)	0.99 (0.94 –1.05)	0.97 (0.9 – 1.04)
Bilirubin	0.92 (0.78 – 1.09)	1.05 (0.91 – 1.2)	1.08 (0.92 – 1.28)
Urea	**1.63 (1.06 – 2.51)***	0.88 (0.48 – 1.62)	1.25 (0.56 – 2.78)
Createnine	1.01 (0.97 – 1.04)	1.00 (0.97 – 1.030	0.95 (0.89 – 1.02) ¶
Alanine transaminase (ALT)	0.95 (0.89 – 1.02) ¶	0.99 (0.94 – 1.03)	1.00 (0.97 – 1.03)
Aspartate aminotransferase (AST)	0.99 (0.95 – 1.03)	1.00 (0.97 – 1.04)	1.00 (0.97 – 1.04)
Preeclampsia	4.36 (0.80 – 23.81) ¶	Omitted	Omitted
Hypertension	0.84 (0.19 – 3.78)	Omitted	Omitted
APGAR 1 min	**0.38 (0.25 – 0.60)*****	**0.40 (0.25 – 0.64)*****	0.79 (0.55 – 1.11) ¶
APGAR 5 min	**0.43 (0.27 – 0.67)*****	**0.47 (0.30 – 0.73)****	0.81 (0.59 – 1.11) ¶
C-section	**4.94 (2.08 – 11.74)*****	**2.87 (1.14 – 7.22)***	1.62 (0.53 – 4.94)
Urinary Tract Infection	0.62 (0.08 – 4.89)	0.57 (0.13 – 2.59)	0.32 (0.04 – 2.48)
Induced labor	**5.55 (1.81 – 16.97)****	**3.36 (1.07 – 10.59)***	1.13 (0.3 – 4.28)
COVID-19	1.1 (0.46 – 2.61)	1.26 (0.49 – 3.26)	0.14 (0.04 – 0.46) ***
Gest age at delivery	**0.50 (0.35 – 0.71)*****	NA	**0.82 (0.69 – 0.97) ***
Birthweight	NA	**1.00 (1.00 – 1.00)*****	**1.00 (1.00 – 1.00) ***

*Indicates *p*<0.05; **indicates *p*<0.01; ***indicates *p*<0.001; ¶ indicates *p*<0.25; low frequency variables were omitted from analysis.

Table S2. Association of ACE2 polymorphisms with maternal and perinatal outcomes. Genotype frequencies and inheritance patterns

**Mode of inheritance**		**Low birthweight (n=24)**			**Preterm birth (n=21)**			**PROM (n=16)**		
		**Case/ Control**	**OR (95% CI)**	**AIC/BIC**	**Case/ Control**	**OR (95% CI)**	**AIC/BIC**	**Case/ Control**	**OR (95% CI)**	**AIC/BIC**
rs2158082	HWE	0.471			0.254			0.339		
Additive	AA	10/123	Ref.	137.79/148.25	10/103	Ref.	112.53/122.56	6/112	Ref.	107.52/117.64
	AG	5/86	0.72 (0.24–2.17)		2/79	0.26 (0.06–1.22)¶		5/80	1.17 (0.34 – 3.96)	
	GG	6/11	6.71 (2.05–21.95)***		6/9	6.87 (2.03–23.26)		4/9	8.3 (1.97 – 34.87) ***	
Dominant	AA	10/123	Ref.	146.08/153.05	10/103	Ref.	126.66/133.34	6/112	Ref.	111.56/118.31
	AG+GG	11/97	1.39 (0.57–3.42)		8/88	0.94 (0.35–2.48)		9/89	1.89 (0.65 – 5.5)	
Recessive	AA+AG	**15/209**	**Ref.**	**136.15/143.12**	**12/182**	**Ref.**	**114.22/120.91**	**11/192**	Ref.	**105.58/112/33**
	GG	**6/11**	**7.6 (2.47– 23.39) *****		**6/9**	**10.11 (3.09–33.13) *****		**4/9**	**7.76 (2.06 – 29.19) *****	
Overdominant	AA+GG	16/134	Ref.	144.58/151.55	16/112	Ref.	119.21/125.89	10/121	Ref.	112.7/119.45
	AG	5/86	0.49 (0.17–1.38)		2/79	0.18 (0.04–0.79) ¶		5/80	0.76 (0.25 – 2.29)	
Allele	A	25/332	Ref.	284.56/292.91	22/285	Ref.	246.49/254.56	15/151	Ref.	164.33/171.12

	G	17/108	2.09 (1.09–4.02)¶		14/97	1.87 (0.92–3.8)		13/41	3.19 (1.41 – 7.24) **	
HWE / rs4830974		0.622			0.592			0.379		
Additive	AA	8/99	Ref.	134.3/144.57	8/84	Ref.	116.76/126.56	4/93	Ref.	107.25/117.22
	AG	6/91	0.82 (0.27– 2.44)		4/78	0.54 (0.16–1.86)		7/84	1.94 (0.55 – 6.85)	
	GG	6/17	4.37 (1.35–14.17) ¶		6/14	4.5 (1.36–14.94) *		4/13	7.15 (1.59 – 32.14)*	
Dominant	AA	8/99	Ref.	138.9/145.75	8/84	Ref.	123.79/130.33	4/93	Ref.	108.43/115.08
	AG+GG	12/108	1.38 (0.54– 3.5)		10/92	1.14 (0.43–3.03)		11/97	2.64 (0.81 – 8.57)	
Recessive	AA+AG	**14/190**	**Ref.**	**132.43/139.28**	**12/162**	**Ref.**	**115.77/122.3**	**11/177**	Ref.	**106.34/112.99**
	GG	**6/17**	**4.79 (1.63–14.07) ****		**6/14**	**5.79 (1.88–17.76) ****		**4/13**	**4.95 (1.38 – 17.73) ***	
Overdominant	AA+GG	14/116	Ref.	137.85 /144.7	14/98	Ref.	120.36/126.9	8/106	Ref.	111.29/117.94
	AG	6/91	0.55 (0.2– 1.48)		4/78	0.36 (0.11–1.13)		7/84	1.1 (0.38 – 3.17)	
Allele	A	22/289	Ref.	271.19/279.43	20/246	Ref.	240.78/248.7	14/131	Ref.	160.71/167.35
	G	18/125	1.89 (0.98– 3.65)		16/106	1.86 (0.93–3.72)		14/45	2.91 (1.29 – 6.57) *	
HWE / rs2285666		0.376			0.325			0.162		

Additive	CC	16/99	Ref.	147.25/157.76	10/91	Ref.	127.61/137.65	9/92	Ref.	119.2/129.36
	CT	4/95	0.26 (0.08– 0.81) ¶		5/78	0.58 (0.19–1.78)		6/82	0.75 (0.26 – 2.19)	
	TT	2/30	0.41 (0.09– 1.9)		3/23	1.19 (0.3–4.67)		1/28	0.37 (0.04 – 3.01)	
Dominant	CC	**16/99**	**Ref.**	**145.5/152.51**	**10/91**	**Ref.**	**126.41/133.11**	9/92	Ref.	117.7/124.46
	CT+TT	**6/125**	**0.3 (0.11–0.79)***		**8/101**	**0.72 (0.27–1.91)**		7/110	0.65 (0.23 – 1.81)	
Recessive	CC+CT	20/194	Ref.	151.84/158.85	15/169	Ref.	126.55/133.24	**15/174**	**Ref.**	**117.49/124.26**
	TT	2/30	0.65 (0.14– 2.91)		3/23	1.47 (0.39–5.47)		**1/28**	**0.41 (0.05 – 3.26)**	
Overdominant	CC+TT	18/129	Ref.	146.81/153.82	13/114	Ref.	125.67/132.36	10/120	Ref.	118.32/125.09
	CT	4/95	0.3 (0.1– 0.92) ¶		5/78	0.56 (0.19–1.64)		6/82	0.88 (0.31 – 2.51)	
Allele	C	36/293	Ref.	295.04/303.44	25/260	Ref.	249.66/257.74	23/128	Ref.	179.00/185.82
	T	8/155	0.42 (0.19– 0.93) ¶		11/124	0.92 (0.44–1.94)		7/66	0.59 (0.24 – 1.45)	

*Indicates $p<0.017$; **indicates $p<0.0085$; ***indicates $p<0.00425$; ¶ indicates $p<0.05$

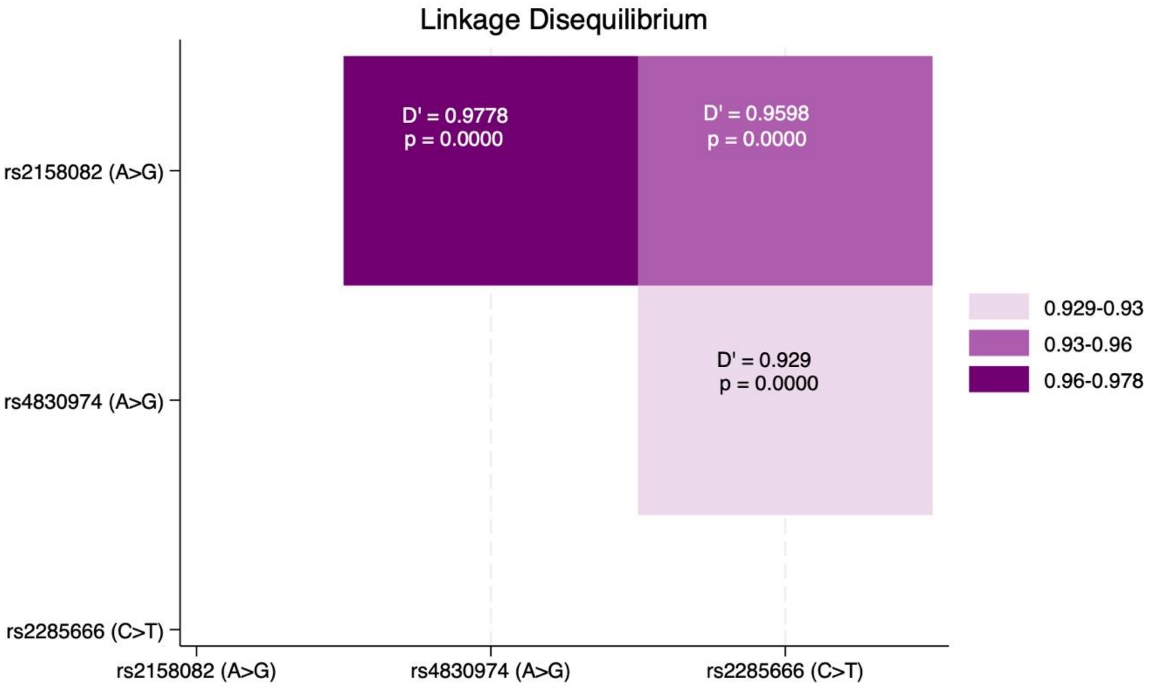

Figure S1. Linkage disequilibrium of the ACE2 SNPs.
